# Supplementary material for: Furanocoumarins as Enhancers of Antitumor Potential of Sorafenib and LY294002 toward Human Glioma Cells In Vitro
Source: Int J Mol Sci. 2024 Jan 7;25(2):759. doi: 10.3390/ijms25020759 (PMC10815922; doi:10.3390/ijms25020759)
Supplement: Supplementary file 1 [file ijms-25-00759-s001.zip › ijms-2801238-supplementary.pdf]

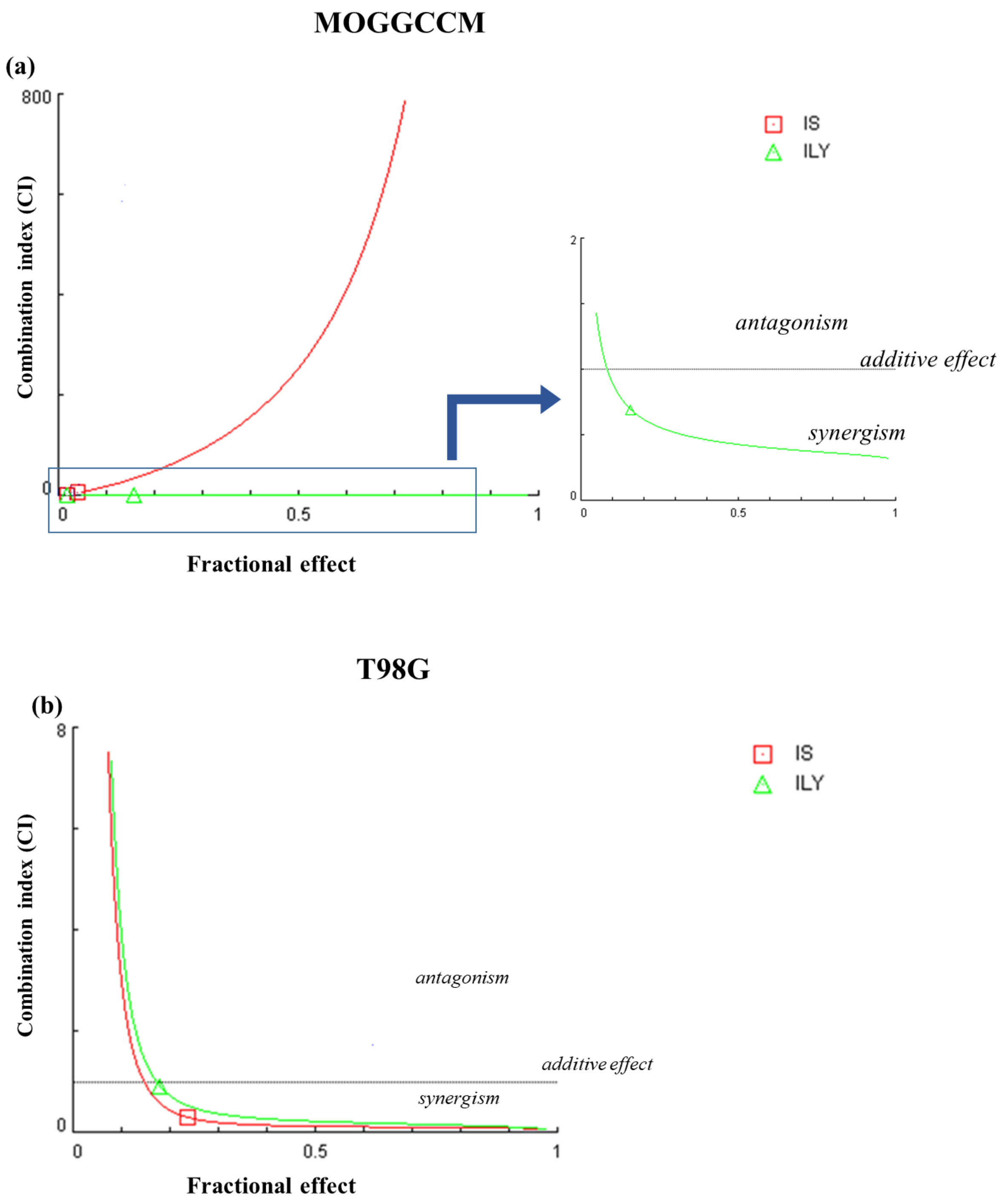

Figure S1 Imperatotin and LY294002 and/or sorafenib combination treatment in MOGGCCM (a) and T98G (b) cells. Combination index (CI) plot: The combination index is plotted as a function of Fa (fractional effect). Combination index (CI) was calculated according to the method of Chou and Talalay using the Compusyn software. CI<1, CI=1 and CI>1 indicate synergistic effect, additive effect, and antagonistic effect, respectively.

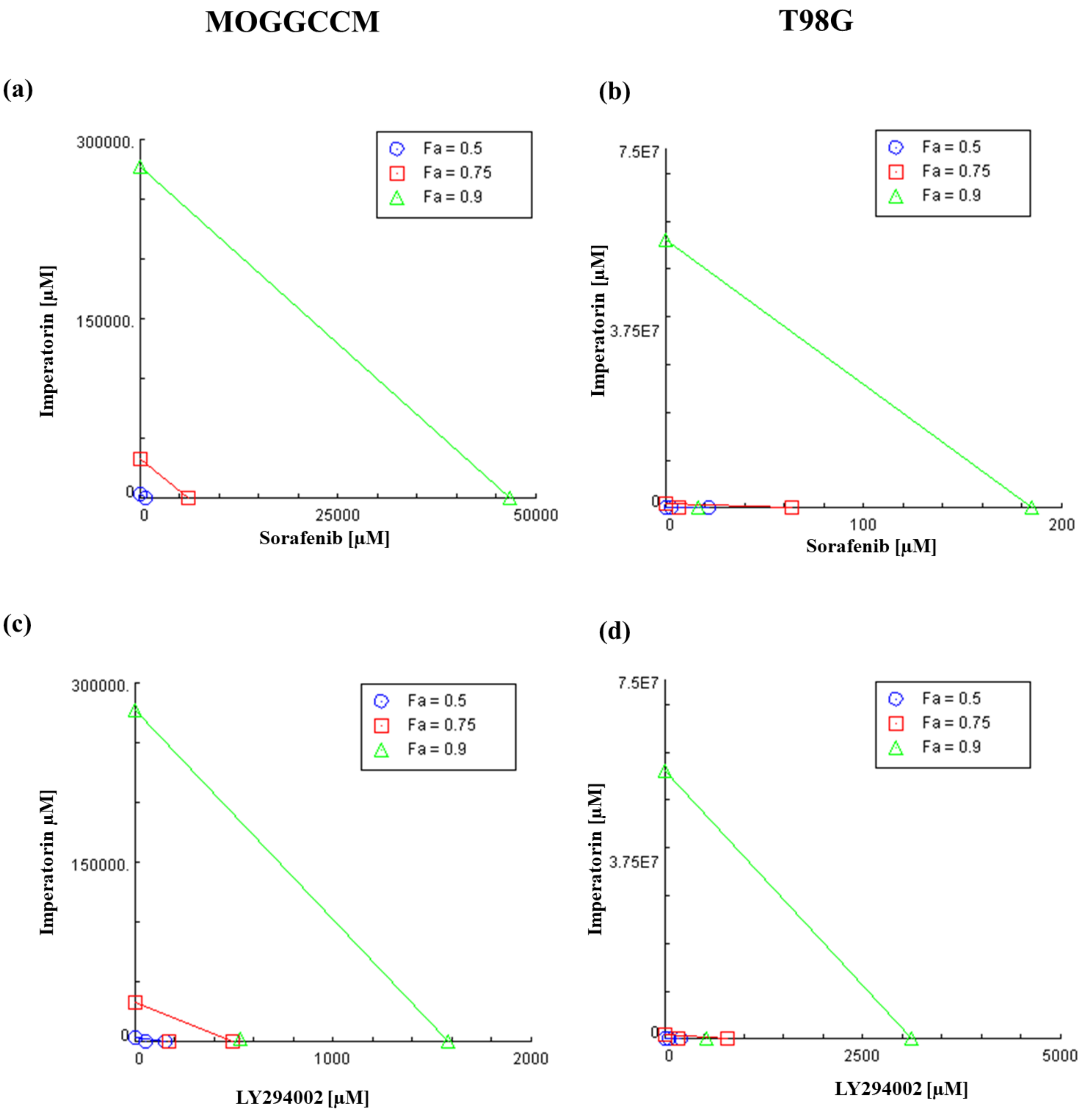

Figure S2 Isobologram for combination of imperatorin and sorafenib (a,b) or LY294002 (c.d) in MOGGCCM (a,c) and T98G (b,d) cell line. Classic isobologram at IC50, IC75, and IC90. (c) the Fa-DRI (dose reduction index) plot (Chou-Martin plot)

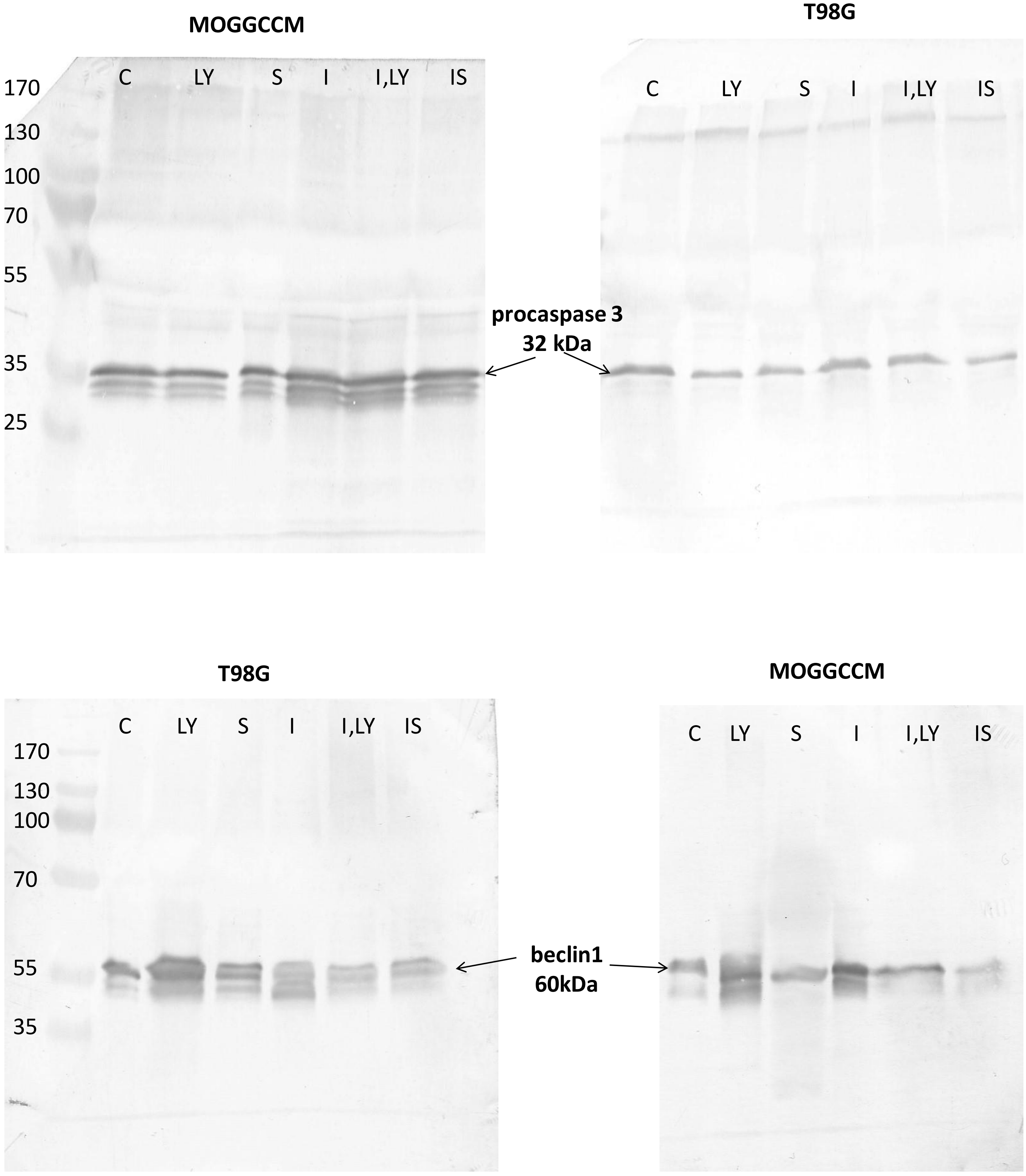

Figure S3 Whole immunoblotting membranes of procaspase 3 and beclin 1 in MOGGCCM and T98G cells. C – control, LY – LY294002, S – sorafenib, I – imperatorin.

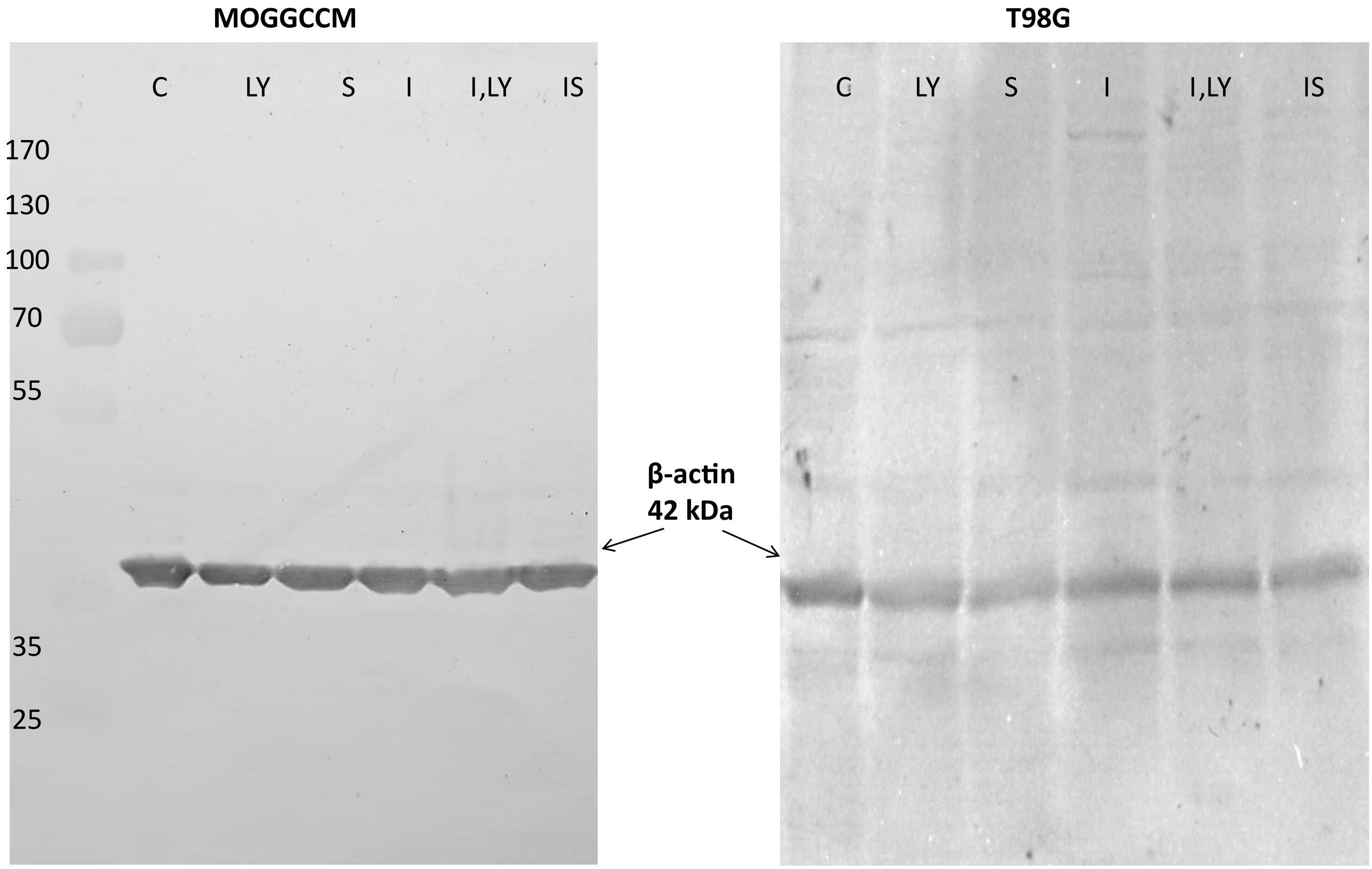

Figure S4 Whole immunoblotting membranes of β-actin in MOGGCCM and T98G cells. C – control, LY – LY294002, S – sorafenib, I – imperatorin.

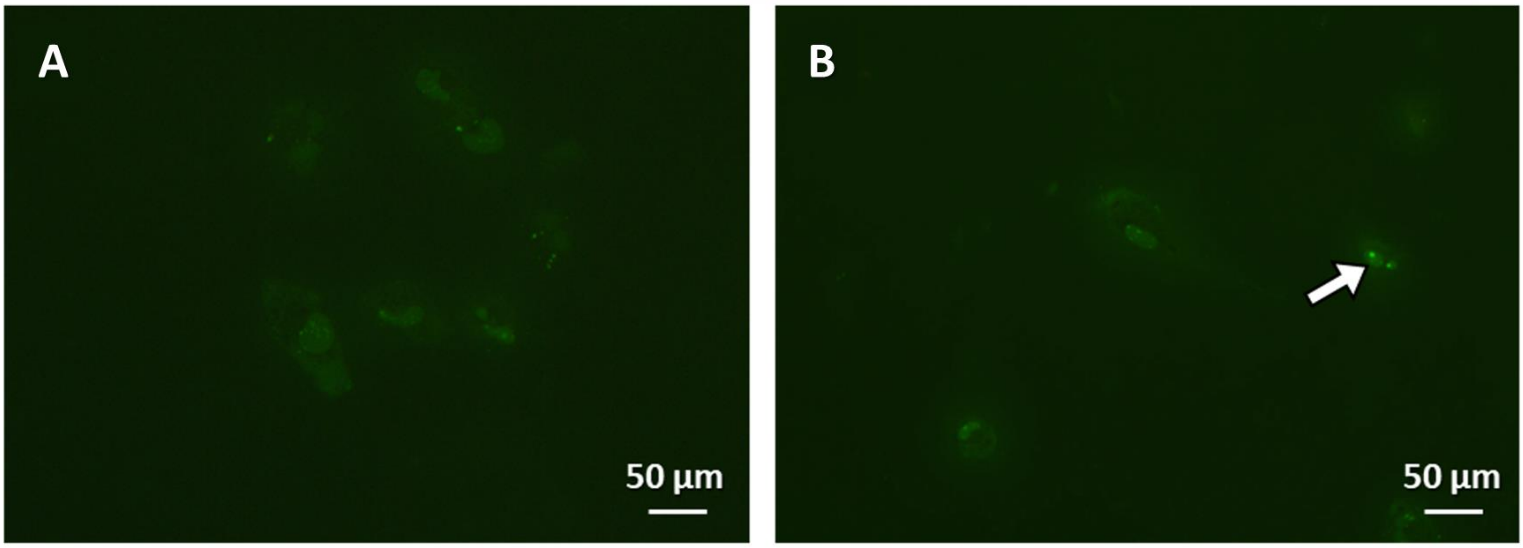

Figure S5 Representative photos of transfection efficiency control in cancer cells. (A) – non - transfected cells, (B) – control siRNA transfected cells (white arrow)
